# The case for plural PBL: an analysis of dominant and marginalized perspectives in the globalization of problem-based learning

**DOI:** 10.1007/s10459-019-09930-4

**Published:** 2019-10-17

**Authors:** Janneke M. Frambach, Wagdy Talaat, Stella Wasenitz, Maria Athina (Tina) Martimianakis

**Affiliations:** 1 School of Health Professions Education, Faculty of Health, Medicine and Life Sciences, Maastricht University, P.O. Box 616, 6200 MD Maastricht, The Netherlands; 2 Medical Education Department, Faculty of Medicine, Suez Canal University, Ismailia, Egypt; 3 Department of Arts and Humanities, Teachers College, Columbia University, New York City, USA; 4 Department of Paediatrics and The Wilson Centre, University of Toronto, Toronto, Canada

**Keywords:** Problem-based learning, Globalization, Contextualization, Culture, Universalism, Neo-colonialism, Health professions education, Medical education, Discourse, Critical narrative review

## Abstract

The globalization of problem-based learning (PBL) in health professions education has been both celebrated and criticized. Using a critical narrative review approach, underpinned by our archive of global PBL literature and a targeted literature search, we analyze these dominant global discourses of PBL in health professions education. More precisely, we explore what is missed when the globalization of PBL is theorized either as a positive consequence of standardization, or a problematic spread of Western educational ideals and values around the world. We make visible how two dominant global discourses, a universalist and culturalist discourse, have emerged in the global proliferation of PBL. We also discuss the limitations of the two discourses by demonstrating how they either ignore contextual and cultural diversity or see it as problematic. We then turn to a perspective that has been marginalized in the PBL literature that emphasizes the global origins of PBL, transcending the dichotomy between West and non-West. We make a case for relating to PBL as a plural construct in order to learn from the cultural and situational nuances of educational activities labeled PBL around the world. We argue that PBL as a singular and universal concept has no global future, yet versions of PBL may continue to thrive locally. Finally, we propose avenues for future research that may help elucidate the global and local values that underpin our curricula, as well as the socio-political factors that perpetuate neo-colonialist views and practices in the uptake and implementation of PBL approaches across the globe.

## Introduction

The globalization of health professions education has resulted in more collaboration between educational institutions and a greater flux of students and teachers across the globe (Hodges et al. 2009; Martimianakis and Hafferty 2013). Indeed, this globalization has facilitated the exchange and sharing of curricula, educational materials, ideas, innovations, technologies, expertise, policies, and standards (Scholte 2005; Hodges et al. 2009; Ho et al. 2015). Whereas many authors have recognized the benefits of globalization in terms of economic, academic, humanitarian, social, and political gains (Altbach and Knight 2007; Bleakley et al. 2008), several important ethical questions have emerged. For example, who benefits from globalization, and who does not (Hodges et al. 2009)? Do those who sell medical education internationally remain critical of their product and its implications across contexts (Whitehead 2016)? How and why are education standards from countries that have a strong international presence in health professions education, often Western countries, imposed globally (Hodges 2016)? In particular, Bleakley et al. (2008) argue that globalization has facilitated the global spread of Western medical education, underpinned by a “set of cultural attitudes that are rarely questioned”, which, according to the authors, carries the risk of a new wave of Western imperialism. Notably, they critique the global expansion of problem-based learning (PBL) as an unquestioned part of an imperialist agenda based on the Western curriculum (Bleakley et al. 2008).

In this paper, using a critical narrative review approach (Greenhalgh et al. 2018), we build on this critical perspective about PBL’s globalization, but also point to the shortfalls of referring to PBL as a singular construct that is part of a Western imperialist agenda. Specifically, we analyze the dominant discourses currently framing the PBL literature to explore what is missed when the globalization of PBL is theorized either as a positive consequence of the standardization of health professions education, or as a problematic spread of Western educational ideals and values. Throughout, we make a case for referring to PBL as a pluralistic construct to learn from the cultural and situational nuances of educational activities labeled PBL around the world.

We define discourse theoretically as the way language, identities, and activities are constructed as naturally occurring truths (e.g. PBL is a good way to teach). In other words, individuals and organizations that embody dominant conversations related to PBL are regarded as legitimate authorities on how to practice PBL, and in turn contribute to the proliferation of these conversations. At least in health professions education, PBL has been a dominant conversation topic worldwide for 50 years, as evidenced by the number of citations related to PBL (Azer 2015). These conversations have contributed to ideas of what counts as legitimate educational knowledge and expertise, such as what is good teaching, what constitutes helpful facilitation and feedback, and what the training of students with cases should look like. As a result, it has also legitimized and raised the profile of people who possess the expertise in these specific areas, and institutions that adopt these practices.

Previous research on the globalization of health professions education distinguishes between discourses around universalism and homogenization (e.g., focusing on similarities between educational contexts) and discourses around culturalism and hybridization (e.g., focusing on differences between educational contexts) (Gosselin et al. 2016; Martimianakis and Hafferty 2013). We argue that these discourses are also dominant in the literature related to PBL. Below, we analyze representative examples of published empirical, review, and perspective studies that relate to PBL’s worldwide spread. In our review of these studies, we first describe the spread of PBL across the world, and the perceived problems and research that have accompanied this global spread. We then discuss how the universalist and culturalist discourses are expressed in these processes, after which we outline what might be missed if we don’t also reflect on the perspectives that these discourses marginalize. Finally, we propose a way forward to relate to PBL in global and local settings.

The articles used in this paper were identified through our archive of the global literature of PBL, accumulated from our previous research and experiences working in PBL environments for health professions education (Frambach 2014; Frambach and Martimianakis 2017; Frambach and Wasenitz 2018; Talaat et al. 1997, 2008). Additionally, we conducted a targeted literature search using the terms global, international, cultural, and cross-cultural in combination with PBL. Only a subset of the papers reviewed have been cited in this paper. We specifically looked for exemplars that reflect the application of the two dominant discourses as described above. To raise new questions, we also identified papers that reflected marginal perspectives related to the spread of PBL. We note that this paper is not an empirical discourse analysis, nor a systematic review; naturally, our educational and research backgrounds have shaped the selection of articles and the topics discussed in this paper. Three of the four authors are or have been involved heavily in PBL practices and research at dominant schools in the global field of PBL approaches, which has shaped both our positive and negative views of different aspects of PBL around the world. Two authors commonly approach research from a critical theoretical perspective, including issues of globalization. We discussed these diverse viewpoints as a team and have built our argument together. We hope our reframing of the globalization of PBL opens a new avenue of thinking and research on this topic.

## A brief overview of the worldwide spread of PBL

The origin of PBL is typically attributed to the medical curriculum launched at McMaster University in Canada in the 1960s. In the 1970s, Maastricht University in the Netherlands and Suez Canal University in Egypt were among the first adopters of PBL—what was then viewed as a pedagogical innovation. At each of these settings, PBL was contextualized to fit the local educational needs, beliefs, preferences, and available resources. Maastricht University, for example, developed a tightly structured format for the tutorial process that fit the high-school leavers in their undergraduate program, whereas McMaster University used a relatively unstructured tutorial process in the post-graduate program that catered to more mature learners (Servant 2016).

Now, 50 years later, PBL has spread widely across health professions education and beyond. This spread is supported through global levers such as the setting of quality and accreditation standards. For example, on a global scale, the revision of medical school curricula to incorporate PBL is classified as one of three “generations of educational reforms [that] characterize progress during the past century” (Frenk et al. 2010). Indeed, global standards for medical education promote curricula that foster students’ self-directed and lifelong learning skills (WFME 2015), which are both central to PBL. Accreditation agencies in several countries have subsequently incorporated PBL curricula as a national accreditation standard, such as in South Korea, Taiwan (Ho et al. 2017), and, for new medical schools, in Ethiopia (Alebachew and Waddington 2015). In several other places, accreditation agencies have strongly encouraged use of PBL-like active learning approaches, such as in Brazil (Bestetti et al. 2014). Hence, PBL has flourished across continents: in Europe, sub-Saharan Africa, Saudi Arabia, Japan, China, and the USA, a majority of medical schools reports to use PBL approaches (AlHaqwi et al. 2015; Chen et al. 2012; Fan et al. 2014; Jippes and Majoor 2008; Kinkade 2005; Saiki et al. 2017).

Over the years, many attempts have been made to define what constitutes PBL. Most published descriptions of PBL include an emphasis on problem cases as a trigger for learning, facilitated small-group work, and independent study (Maudsley 1999; Taylor and Miflin 2008; Wood 2003). There is no consensus on whether PBL is a curriculum-wide strategy or a teaching method to be applied to a single course (Koh 2016; Taylor and Miflin 2008). As a result, some have classified PBL into different types, ranging from PBL as “window-dressing” when it appears in a few sessions across a curriculum, to “full-fledged” PBL curricula when it is used as the primary mode of organizing and delivering content (Nadarajah et al. 2016). Indeed, PBL is used to label a variety of ways to deliver education. For instance, several Asian universities introduced so-called “hybrid curricula” that consist of a combination of lectures, tutorials labeled as PBL, and other instructional methods (Amin et al. 2005; Gwee and Tan 2001; Lam et al. 2006; Yan et al. 2017). In other regions as well, pedagogical approaches labeled PBL have often been used in combination with or as an “add-on” to other approaches, either in separate courses or curricula as a whole (Kinkade 2005; Talaat et al. 2008). Several African universities emphasize community-based education and combine this with what they label as a PBL approach (Iputo 2008; Kiguli-Malwadde et al. 2006; Mullan et al. 2011; Osborne and Needham 2000). Other universities around the world have also experimented with configurations such as integrating virtual patients in PBL seminars (Hamdy et al. 2017), integrating real patients (e.g. at Maastricht University), or having students construct PBL cases (Rustici et al. 2017).

## Problematics and research accompanying the worldwide spread of PBL

Contrary to what the overwhelming uptake of approaches labeled as PBL worldwide may suggest, this uptake process was, and remains, problematic. Wherever a form of PBL is introduced for the first time, there is generally strong resistance against the new approach, including in the Dutch and Egyptian contexts in the 1970s when Maastricht and Suez Canal universities introduced the innovation. Local and national health professions communities in these places, as well as the educational staff, have often raised doubts as to whether PBL will deliver competent graduates compared with traditional, lecture-based curricula. As a result, the field of PBL research was born: worldwide, countless studies have been conducted to investigate whether PBL delivers ‘superior’ graduates compared to lecture-based curricula, such as in the Netherlands (Prince et al. 2005), Mozambique (Frambach et al. 2015), and China (Yan et al. 2017; Zhou et al. 2016). Many of these studies ascribe a certain degree of superiority to PBL, particularly as a superior instructional approach for training competencies in the social and cognitive domains, which review studies echo (Koh et al. 2008; Neville 2009). On the contrary, studies from a critical perspective question the potential of PBL to deliver reflexive, patient-centered and community-oriented graduates (Cavanagh et al. 2019; MacLeod 2011; Milligan 1999).

Once institutions around the world made the shift to some version of PBL, other concerns have commonly come up. The financial costs, physical requirements, demands on human resources, and planning and organization involved in implementing and sustaining PBL curricula are listed as obstacles and challenges, particularly in, but not limited to, the applicability of PBL in low-income countries (Amin et al. 2005; Camp 1996; Carrera et al. 2003; McLean 2004; Mufunda et al. 2007; Shanley 2007; Talaat et al. 2008). Following these concerns, the global PBL research field produced a wave of studies on the role of contextual factors in PBL implementation and students’ learning experiences. Case studies on the successes, challenges, and results of PBL implementation were conducted in several parts of the world, including Japan (Oda and Koizumi 2008), the Netherlands (Moust et al. 2005), Singapore (Gwee and Tan 2001), South Africa (Kwizera et al. 2005), Qatar (Nasr and Wilby 2017), and Uganda (Kiguli-Malwadde et al. 2006). Overview studies summarized insights on students’ learning experiences in PBL, the role of context, and how to keep the approach effective (Bate et al. 2014; Dolmans et al. 2005, 2016; Lim 2012; Wood et al. 2015).

The cross-cultural applicability of PBL as an educational practice developed in Canada—with a Western context—has been challenged repeatedly by scholars (Bleakley et al. 2008). For example, scholars have argued that specific ways of verbal expression and collaborative and self-directed learning expected of students in PBL settings might not fit with particular educational and socio-cultural contexts (Frambach 2014). As a result, in many non-Western parts of the world, the role of cultural factors in PBL pedagogy has become a specific research focus. For example, Gwee (2008) analyzed how Asian communication styles influence PBL processes in Asia; Ju et al. (2016), Al-Kloub et al. (2014) and Zaduqisti (2016), respectively, investigated Korean, Jordanian, and Indonesian students’ perceptions of PBL and the influence of cultural factors; and Frambach et al. (2012, 2014) examined the influence of cultural factors on self-directed learning and small group discussion processes in PBL in the Middle East, East Asia and Europe. Some of these studies position cultural factors as challenges to be overcome (e.g. Frambach et al. 2012; Ju et al. 2016), whereas others approach them as aspects that can both impede and facilitate PBL processes (e.g. Gwee 2008; Zaduqisti 2016). Together, these studies suggest that cultural factors shape PBL processes, experiences, and outcomes, and therefore these cultural factors need to be carefully considered in PBL implementations. These studies further argue that PBL needs to be adapted to the local and institutional socio-cultural context; for example, in relation to approaches towards assessment and how tutors guide PBL tutorials. This may partly explain the different forms that PBL has taken worldwide. Additionally, some authors conclude that alternative pedagogical approaches other than PBL should be developed if they better fit the local context than PBL (e.g. Frambach et al. 2012). In contrast, other authors contend that local students and staff must adapt to PBL by changing their mindset to better fit with PBL (e.g. Ju et al. 2016).

## Two dominant global discourses of PBL: universalism and culturalism

From the literature reviewed above, we discern a universalist and a culturalist discourse related to the globalization of PBL. The universalist discourse describes PBL as a ‘singular’ concept; as an educational approach that can be, and ideally should be, implemented everywhere in more or less similar ways. This discourse is based on and supported by: the overwhelming worldwide uptake of PBL, the integration of PBL in global standards and national accreditation frameworks, and the large body of PBL research that seeks to establish a definition. In short, this perspective trumpets PBL’s superiority over other approaches and argues that students and staff across various contexts need to adapt to the approach. Conversely, the culturalist discourse operationalizes PBL as a ‘plural’ concept; as an educational approach that reflects the cultural norms and values of the context where it was developed, and that therefore should be adapted to contexts where it is implemented. This discourse is based on and supported by: the worldwide contextualization and differentiation of PBL across institutions, research that investigates the role of culture and context in PBL implementation, the lack of consensus on PBL definitions, and the need to adapt PBL to fit the specific context or else develop an alternative pedagogical approach that better fits the context.

In both cases, the notion of a ‘PBL expert’ is not challenged. Indeed, both discourses converge on the idea that there is expertise associated with implementing and sustaining PBL, either in the form of a ‘golden standard’, or in developing culturally sensitive versions of PBL. These ideas also extend to those who evaluate PBL curricula and initiatives (e.g., in relation to accreditation and funding opportunities). In practice, this enables institutions “who have historically been in a position to build capacity and experience in PBL and share their knowledge and research worldwide, such as Maastricht University, to continue being looked at for expertise (…), possibly at the expense of others who did not have such a head start or who have a different educational message that is less aligned with mainstream global discourse” (Frambach and Martimianakis 2017).

Moreover, both discourses maintain that PBL originated in the West. In the universalist case, however, the mentality seems to be that the approach has global applicability and others need to assimilate, whereas in the culturalist case the mentality seems to be that others need to adapt PBL or else create an alternative that might not have the same status as the ‘original’ PBL, but that would still be similar to it. Therefore, we argue that the two dominant global discourses of PBL perpetuate neo-colonialist views and practices in health professions education globally, such that those who possess expertise of what are considered Western-developed methods hold the power to define the conditions for the globalization of PBL and control its implementation and evolution over time. These subtle yet major effects of these dominant discourses are recognizable through the “superhero” or “iconic” status that PBL gained in health professions education, with schools worldwide eagerly joining the PBL movement—some for reasons of publicity and competitiveness (i.e., obtaining accreditation or attracting students) rather than educational effectiveness per se (Nadarajah et al. 2016).

## A marginalized perspective: the global origins of PBL

A possible way to transcend such power relations can be found in one perspective that seems often marginalized or neglected in these discussions. As many of the studies discussed above demonstrate, PBL becomes something different across various institutions. In its practice worldwide, PBL is not a Western concept, despite often being regarded as such. The term was coined in Canada in the 1960s, but what is often not acknowledged when referring to the roots of PBL are the global origins of these beliefs and practices. Though the invention of PBL is both claimed by and ascribed to the West, the values behind PBL elements such as collaborative and student-centered learning can be traced to the distant past and across many different cultures. For example, Lee et al. (2004) describe how PBL converges with the educational philosophy of various ancient Chinese schools, including Confucianism, neo-Confucianism, and Taoism, which emphasize integration of knowing and doing, critical reflection and debate, individuality of learning, self-motivated learning, timeliness of instruction, and cooperative learning. Similarly, Saiki et al. (2017) note how the Japanese concepts of “hansei” and “kaizen” resemble the concepts of critical reflection and self-directed action, which are often expected of students in PBL settings. Kombar (1994) also describes how a shift from traditional to student-centered education characterized pre-PBL twentieth-century education in Egypt under the leadership of Egyptian education scholar Al-Qabbani. Additionally, collaborative learning in the form of groups was part of many ancient societies, from India to Greece and beyond.

In previous globalization waves, these educational practices and beliefs travelled the globe in a “centuries-long, multidirectional history of sharing” (Whitehead et al. 2018). The relation that is often made between the development and enactment of PBL and the works of mainly Western philosophers, such as Dewey, Marx, Popper, Socrates, as well as the Brazilian philosopher Freire (Servant 2016; Wang et al. 2008) may therefore not be the only relevant educational philosophies for thinking about PBL. In addition to Servant and Schmidt’s (2016) statement that these intellectual roots of PBL are in fact not as strong and clear-cut as some portray, we argue that PBL’s roots are more global and complex than is reflected by its current dominant portrayal as a Western concept.

This perspective complicates the distinction between ‘Western’ and ‘non-Western’. It also complicates statements made by critical, post-colonial scholars of globalization in health professions education—including authors of this paper—who argue that the spread of PBL can pose a threat to cultural education values in countries that import the approach as a way to meet accreditation standards or to enhance the quality and caliber of their curricula (Bleakley et al. 2008; Frambach et al. 2012; Hodges et al. 2009). In some cases, the spread of PBL may reflect the opposite: shifting away from the colonial and neo-colonial Euro-American education systems that were established in many health professions education institutions in Asia and sub-Saharan Africa during the nineteenth and early twentieth centuries (Whitehead et al. 2018) to approaches that might better align with local ideas about how to support learning. Of course, this assertion must be supported empirically with research that takes into consideration the power relations at play in the spread of PBL thus far.

What we propose is to no longer define PBL as a purely Western concept, and instead we should recognize that PBL is not one thing, and thus that no one can ever ‘own’ the concept. Although the various definitions, interpretations, and implementations of PBL have been emphasized previously, the dominant global discourses of PBL tend to either ignore this diversity or see it as a problem to be overcome. Embracing the potential afforded by the multiple guises of PBL, without a universal standard in mind, and acknowledging that all answers to the question “what does PBL mean to you?” are legitimate, may enable the field of health professions education to move towards more equitable educational practices and research conversations.

## Moving forward: PBL as plural and local

What, then, is the future of PBL as ‘not one thing’, and how can we embrace diversity rather than see it as problematic? We argue that PBL’s future is mainly local, so institutions must decide internally how best to approach their education. In an ideal world this would happen without direct or indirect pressure from dominant players in the field (e.g. those who control the accreditation of new schools and the publication of research) and instead be shaped by the global dissemination of best practices, underpinned by research on the science of learning. PBL, as a term, has come to mean many different things. It is often used simply as a short form to signify an appreciation for the idea that learning can be self-directed and can move away from large group lectures. For this reason, each institution may find it useful to define whether their approach is reminiscent of PBL, how they interpret PBL, and how they shape and implement it locally. At a global level, institutions may share their experiences with other institutions as part of a global conversation for improving education. We believe this ultimate goal of improving education is best served if this conversation includes participants bringing in a rich diversity of educational approaches and perspectives, and focuses on how we can deliver high quality graduates rather than how we label our approach or who is allowed to use that label. This means recognizing and encouraging the plurality of approaches that has developed, not only as part of the PBL movement, but as part of student-centered and community-oriented education more broadly. To be effective, power relations must also be considered when organizing and participating in global sharing events and practices, considering that those currently in power have more access to resources, and greater capacity to control how we disseminate and share knowledge about PBL approaches.

Thus, the term PBL as a singular and universal concept has no global future. Globally, we can only talk about PBL in the plural sense, which we may emphasize by using plural expressions such as ‘PBL approaches’ or ‘PBL strategies’. This subtle change in terminology has implications for how the term is currently used worldwide (e.g., in the context of accreditation standards, consultation services, and research questions) because these situations often require a singular, shared understanding across contexts and institutions. Rather than “nailing down definitions”, the potential value of exploring the meaning of educational concepts, including the philosophies and values that underpin them in different contexts worldwide, has been emphasized previously in terms of opening up alternative ways of seeing and knowing what may be better in other contexts (Naidu and Kumagai 2016). In other words, if we see this diversity as an opportunity to invest resources into more scientific explorations that focus on understanding what educational modality works in what contexts and why (Dolmans and Gijbels 2013), we may be surprised by what benefits it yields for our local practice. Importantly, this stance may put more power in the hands of low-resource or non-Western countries, perhaps contributing to a more equitable conversation about what better educational outcomes look like.

In addition to functioning as a platform for sharing local experiences and interpretations of PBL, how and why it might function in different contexts, and opening up to approaches of student-centered education more broadly, research on PBL approaches may focus on the factors that draw institutions worldwide to constructs of PBL. Specifically, future research should analyze why people and institutions are drawn to PBL constructs, what they see as added value of using the term, how they take decisions regarding its adoption and implementation, and what forces guide these decisions. These avenues of research may help elucidate the global and local values that underpin our curricula, as well as the socio-political factors that perpetuate neo-colonialist views and practices in the uptake and implementation of PBL approaches across the globe.
